# Towards a unified protocol for handling of CSF before β-amyloid measurements

**DOI:** 10.1186/s13195-019-0517-9

**Published:** 2019-07-19

**Authors:** Shorena Janelidze, Erik Stomrud, Britta Brix, Oskar Hansson

**Affiliations:** 1 Department of Clinical Sciences Malmö, Clinical Memory Research Unit, Lund University, Sölvegatan 19, BMC B11, 221 84 Lund, Sweden; 2 Memory Clinic, Skåne University Hospital, Simrisbanvägen 14, SE-20502 Malmö, Sweden; 3 EUROIMMUN AG, 23560 Luebeck, Germany

**Keywords:** Cerebrospinal fluid, Biomarkers, Pre-analytical variables, Alzheimer’s disease diagnosis, β-Amyloid

## Abstract

**Background:**

Widespread implementation of Alzheimer’s disease biomarkers in routine clinical practice requires the establishment of standard operating procedures for pre-analytical handling of cerebrospinal fluid (CSF).

**Methods:**

Here, CSF collection and storage protocols were optimized for measurements of β-amyloid (Aβ). We investigated the effects of (1) storage temperature, (2) storage time, (3) centrifugation, (4) sample mixing, (5) blood contamination, and (6) collection gradient on CSF levels of Aβ. For each study participant, we used fresh CSF directly collected into a protein low binding (LoB) tube that was analyzed within hours after lumbar puncture (LP) as standard of truth. Aβ42 and Aβ40 were measured in de-identified CSF samples using EUROIMMUN and Mesoscale discovery assays.

**Results:**

CSF Aβ42 and Aβ40 were stable for at least 72 h at room temperature (RT), 1 week at 4 °C, and 2 weeks at − 20 °C and − 80 °C. Centrifugation of non-blood-contaminated CSF or mixing of samples before the analysis did not affect Aβ levels. Addition of 0.1–10% blood to CSF that was stored at RT without centrifugation led to a dose- and time-dependent decrease in Aβ42 and Aβ40, while Aβ42/Aβ40 did not change. The effects of blood contamination were mitigated by centrifugation and/or storage at 4 °C or − 20 °C. Aβ levels did not differ between the first to fourth 5-ml portions of CSF.

**Conclusions:**

CSF can be stored for up to 72 h at RT, 1 week at 4 °C, or at least 2 weeks at either − 20 °C or − 80 °C before Aβ measurements. Centrifugation of fresh non-blood-contaminated CSF after LP, or mixing before analysis, is not required. In case of visible blood contamination, centrifugation and storage at 4 °C or − 20 °C is recommended. After discarding the first 2 ml, any portion of up to 20 ml of CSF is suitable for Aβ analysis. These findings will be important for the development of a clinical routine protocol for pre-analytical handling of CSF.

**Electronic supplementary material:**

The online version of this article (10.1186/s13195-019-0517-9) contains supplementary material, which is available to authorized users.

## Background

Cerebrospinal fluid (CSF) Alzheimer’s disease (AD) biomarkers, β-amyloid 42 (Aβ42) and tau, have been incorporated into the clinical research criteria for AD diagnosis proposed by the US National Institute on Aging–Alzheimer’s Association (NIA-AA) [1] and International Working Group (IWG) for New Research Criteria for the Diagnosis of AD [2] However, to be able to implement CSF AD biomarkers worldwide, we need to have (i) appropriate use criteria in place, (ii) high precision methods to determine CSF AD biomarker levels with very low within and between laboratory variations, and (iii) a unified pre-analytical protocol for handling of CSF before analyses. Recently, the Alzheimer’s Association multidisciplinary workgroup released criteria for the appropriate use of lumbar puncture (LP) and CSF testing in the diagnosis of AD to facilitate decision-making by healthcare practitioners [3]. Further, several commercially available immunoassays measuring CSF AD biomarkers have been optimized resulting in very high precision for the determination of AD biomarkers [4, 5]. Ongoing effort to produce certified reference materials [6] and recent development of a certified mass spectrometry (MS)-based reference measurement procedure for Aβ42 [7] have already harmonized biomarker data obtained using different assays. At the same time, a significant proportion of the variability in reported AD biomarker levels between centers is due to the differences in pre-analytical procedures, and therefore, the standardization of biomarker measurements and establishment of global cutoffs will critically depend on the development of a unified pre-analytical protocol. Previous investigations have shown that multiple pre-analytical factors may influence CSF levels of AD biomarkers [8]. For example, differences in collection tube types, storage temperature, and duration; handling of blood-contaminated samples; centrifugation steps; and the number of freeze-thaw cycles may introduce more than 50% change in CSF Aβ42 concentrations [8–11]. However, previous studies were confined to the analysis of samples that were stored frozen after collection and thus lack a proper standard of truth, i.e., fresh CSF obtained directly in protein low binding (LoB) tubes and analyzed after LP without any pre-analytical steps in-between collection and analyses. Further, a protocol developed for use in clinical practice needs to be as simple as possible to facilitate implementation worldwide.

The aim of the present study was to study the effects of different pre-analytical handling procedures using fresh CSF analyzed within hours after LP as the standard of truth, paving the way for a unified pre-analytical protocol for clinical routine use. We chose to focus on CSF Aβ measurements, because earlier findings suggest that amyloid peptides, Aβ42 and Aβ40, are particularly sensitive to pre-analytical handling whereas tau is less affected [12–15]. We investigated how pre-analytical factors (including storage at different temperatures and for different periods of time, centrifugation, mixing of samples before Aβ analysis, and blood contamination) influence CSF levels of Aβ42 and Aβ40. To limit potential effects of analytical variables, Aβ42 and Aβ40 were measured using 2 different immunoassays, EUROIMMUN (EI) and Mesoscale discovery (MSD).

## Materials and methods

### CSF collection

De-identified CSF samples from a total of 59 patients were used in this study. Patients were undergoing LP as a part of the Swedish BioFINDER Study or due to clinical suspicion of normal pressure hydrocephalus at the Memory Clinic, Skåne University Hospital, Sweden. All patients gave their written informed consent allowing their CSF samples to be used for research. Previous studies have shown that compared to polypropylene, LoB material significantly reduces Aβ42 loss due to adsorption to plastic [11, 16]. Therefore, LoB tubes were used for CSF collection. In all pre-analytical protocols, after discarding the first 2 ml, 0.5–0.75 ml of CSF was dripped from a LP needle directly into 1.5-ml LoB tubes (Sarstedt, Nümbrecht, Germany, catalog number 72.703.600). The number of tubes per each study participant and the treatment and analysis of CSF samples in different pre-analytical protocols are described below.

### Pre-analytical protocols

#### CSF storage at different temperatures

##### Room temperature and 4 °C

CSF was collected from 12 patients (3 patients per day, 10 CSF tubes per patient). From all 12 participants, all 10 CSF samples were analyzed within hours (h) after LP (baseline; for flowchart, please see Additional file 1: Figure S1A). After baseline analysis, 5 CSF tubes from each patient were stored refrigerated at 4 °C and the other 5 tubes at room temperature (RT; 19–23 °C). Two CSF samples from each patient (1 tube that had been stored at RT and the other stored at 4 °C) were analyzed at 24 h, 48 h, 72 h, 1 week, or 2 weeks after collection. CSF from each tube was analyzed twice, i.e., at baseline and at one of the five different time points, 24 h, 48 h, 72 h, 1 week, and 2 weeks after collection. These non-frozen CSF samples were not centrifuged after LP. CSF Aβ42 and Aβ40 were measured using EI and MSD kits.

##### –20 °C and −80 °C

CSF was collected from other 10 patients (6 and 4 patients per day, 4 CSF tubes per patient). From all 10 participants, all 4 CSF samples were analyzed at baseline (Additional file 1: Figure S1B). After baseline analysis, 2 CSF tubes from each patient were stored at − 20 °C and the other 2 tubes at − 80 °C. All 4 CSF samples from each patient (2 tubes stored at − 20 °C and the other 2 tubes stored at − 80 °C) were analyzed 2 weeks after collection. Frozen samples were thawed at RT and mixed for 15 min using a roller mixer immediately before the analysis except for few CSF samples that were analyzed without mixing (Additional file 1: Figure S1B). CSF was not centrifuged after LP. Fresh CSF samples were not mixed before the analysis. CSF Aβ42 and Aβ40 were measured using EI and MSD kits.

#### Mixing of CSF before the analysis

CSF was collected from 6 patients (3 patients per day, 4 CSF tubes per patient). From all 6 participants, all 4 CSF samples were analyzed at baseline (Additional file 1: Figure S2). After baseline analysis, 2 CSF tubes from each patient were stored at RT and the other 2 CSF tubes at 4 °C. Two CSF samples from each patient (1 tube stored at RT and the other stored at 4 °C) were mixed for 15 min using a roller mixer immediately before the analysis; the other 2 CSF samples were analyzed without mixing. In addition to baseline, CSF samples from all 4 tubes were analyzed at 24 h, 48 h, 72 h, 1 week, and 2 weeks after collection. CSF was not centrifuged after LP. CSF Aβ42 and Aβ40 were measured using EI and MSD kits.

#### Blood contamination and centrifugation

In the initial experiments (Additional file 1: Figure S3), we studied the effects of blood contamination. CSF was collected from 4 patients (2 patients per day, 12 CSF tubes per patient). For all 4 patients, blood was drawn into serum BD Vacutainer® tubes (Becton Dickinson AB, Stockholm, Sweden) and added to 8 CSF tubes from the same individual (final volume 0.1% [4 CSF tubes] or 10% [4 CSF tubes]) immediately after CSF collection, whereas the remaining 4 tubes contained neat CSF (see Additional file 1: Figure S4 for photographs of blood-contaminated CSF samples). Blood contamination at 0.1% and 10% corresponds to approximately 5000 and 500000 erythrocytes per microliter (E/μl), respectively. A total of 6 tubes per patient (2 neat CSF tubes, 2 CSF-0.1%-blood tubes, and 2 CSF-10%-blood tubes) were centrifuged within 2 h after collection (2000*g*, 10 min). The other 6 tubes (2 neat CSF tubes, 2 CSF-0.1%-blood tubes, and 2 CSF-10%-blood tubes) were not centrifuged. From all 4 participants, all 12 CSF tubes were analyzed on the day of collection. After analysis, CSF and CSF-blood samples (centrifuged and non-centrifuged) were stored at either RT or 4°; one tube of neat CSF, CSF-0.1%-blood, and CSF-10%-blood were used per each four conditions: (i) no centrifugation and storage at RT, (ii) no centrifugation and storage at 4 °C, (iii) centrifugation and storage at RT, and (iv) centrifugation and storage at 4 °C (Additional file 1: Figure S3). In addition to baseline, CSF samples from all 12 tubes were analyzed at 1 week and 2 weeks after collection. Samples were mixed for 15 min using a roller mixer immediately before the analysis. CSF Aβ42 were measured using EI kits.

In the second set of experiments, we studied the effects of blood contamination in more detail. The protocol was the same as for the initial experiment (Additional file 1: Figure S3) with the following modifications. CSF was collected from 7 to 10 patients (1–2 patients per day, 24 CSF tubes per patient) per experimental condition, and we added 1% blood contamination (final volume 0.1%, 1%, or 10%, Additional file 1: Figure S4) and storage at − 20 °C. For − 20 °C, all CSF samples were analyzed at baseline and 2 weeks after collection. Two CSF tubes were used per each condition (centrifugation, blood contamination, and temperature) with all samples and analysis shown in Additional file 1: Figure S5. CSF Aβ42 and Aβ40 were measured using MSD kits.

Finally, we assessed the effect of blood contamination at very low levels on CSF Aβ levels. We used CSF samples with 0.01%, 0.02%, and 0.04% blood which corresponds to approximately 500 E/μl, 1000 E/μl, and 2000 E/μl, respectively (see Additional file 1: Figure S4 for photographs of blood-contaminated CSF samples). CSF was collected from 4 patients (on the same day, 8 CSF tubes per patient). For all 4 patients, blood was drawn into serum BD Vacutainer® tubes (Becton Dickinson AB, Stockholm, Sweden) and added to 6 CSF tubes from the same individual (final volume 0.01% [2 CSF tubes], 0.02% [2 CSF tubes], and 0.04% [2 CSF tubes]) immediately after CSF collection, whereas the remaining 2 tubes contained neat CSF (Additional file 1: Figure S6). A total of 4 tubes per patient (1 neat CSF, 1 CSF-0.01%-blood, 1 CSF-0.02%-blood, and 1 CSF-0.04%-blood tubes) were centrifuged within 2 h after collection (2000*g*, 10 min). The other 4 tubes (1 neat CSF, 1 CSF-0.01%-blood, 1 CSF-0.02%-blood, and 1 CSF-0.04%-blood tubes) were not centrifuged. From all 4 participants, all 8 CSF tubes were analyzed on the day of collection. After analysis, CSF and CSF-blood samples (centrifuged and non-centrifuged) were stored at RT (Additional file 1: Figure S6). In addition to baseline, CSF samples from all 8 tubes were analyzed at 24 h, 72 h, and 1 week, after collection. Samples were mixed for 15 min using a roller mixer immediately before the analysis. CSF Aβ42 were measured using EI kits.

#### CSF collection gradient

CSF was collected from 10 patients. For each patient, four 5-ml portions (P1-P4) of CSF were collected into 5-ml LoB tubes (Eppendorf Nordic A/S, Hørsholm, Denmark) after discarding the first 2 ml. CSF samples were centrifuged after LP (2000*g*, 10 min), and 1 ml was aliquoted into 2-ml LoB tubes (Sarstedt AG & Co., Nümbrecht, Germany) followed by storage at − 80 °C. CSF Aβ42 and Aβ40 were measured in P1–P4 portions using EI and MSD kits.

### Aβ measurements

CSF samples were analyzed using EI Aβ42 and Aβ40 kits and MSD V-plex Aβ peptide panel kit (6E10) and according to the manufacturer’s recommendations. All samples within the same pre-analytical protocol were analyzed using the same batch of the kits. The time delay between LP and the start of the baseline sample analysis was 2–4 h. In MSD runs, samples were analyzed in singlicate because MSD V-plex kit has consistently shown low intra-plate coefficient of variance (Aβ42 CV < 4.3%, Aβ40 CV < 10%) in our previous analyses. For EI kit, all samples were analyzed in duplicate. EI and MSD assays were run in parallel. Each plate included 2 quality control (QC) samples (frozen aliquots of low- and high-concentration Aβ42 or Aβ40 provided in the respective EI kits and 2 pooled CSF samples for MSD assays) that were analyzed in duplicate.

### Statistical analyses

SPSS version 22 (IBM, Armonk, NY, USA) and R version 3.4.3 (RStudio) [17] were used for statistical analysis. When two CSF tubes from individual study participants were included per experimental condition, the mean biomarker concentrations were used in statistical analysis. Changes in the biomarker levels were analyzed with a mixed-effects model including participant identification as a random effect and treatment groups (temperature, centrifugation, and/or blood contamination), time, and time × treatment group interactions as fixed factors. Marginal and conditional *R*^2^ values were computed using the method described by Nakagawa and Schielzeth [18]. To further examine significant interactions, simple main effects analysis and least significant difference post hoc test were performed. The average inter-assay CVs for QC samples were 4% for EI Aβ42 (4.4% and 3.6% for individual QCs) and Aβ40 (3.4% and 4.1% for individual QCs), 9% for Aβ42^MSD^ (8.7% and 9.1% for individual QCs), and 11% for Aβ40^MSD^ (10.8% and 11.7% for individual QCs) assays. Aβ concentrations in the same QC samples analyzed on different plates varied on average by approximately 5% and 10% for EI and MSD assays, respectively. Consequently, for changes in the biomarker levels within these ranges, it was not possible to determine whether they were caused by inter-plate variability or pre-analytical factors. Therefore, only changes exceeding 5% for EI assays and 10% for MSD assays were considered to be due to pre-analytical sample handling and tested in statistical analysis.

## Results

In CSF samples from all study participants analyzed on the day of collection, the median (range) values for Aβ42^EI^, Aβ40^EI^, Aβ42/Aβ40^EI^, Aβ42^MSD^, Aβ40^MSD^, and Aβ42/Aβ40^MSD^ were 747 pg/ml (353–1892 pg/ml), 8343 pg/ml (3541–12933 pg/ml), 0.13 (0.05–0.23), 511 pg/ml (198–1554 pg/ml), 6249 pg/ml (2813–12094 pg/ml), and 0.11 (0.04–0.18), respectively (Additional file 1: Figure S7).

### CSF storage at different temperatures

**Fig. 1:**
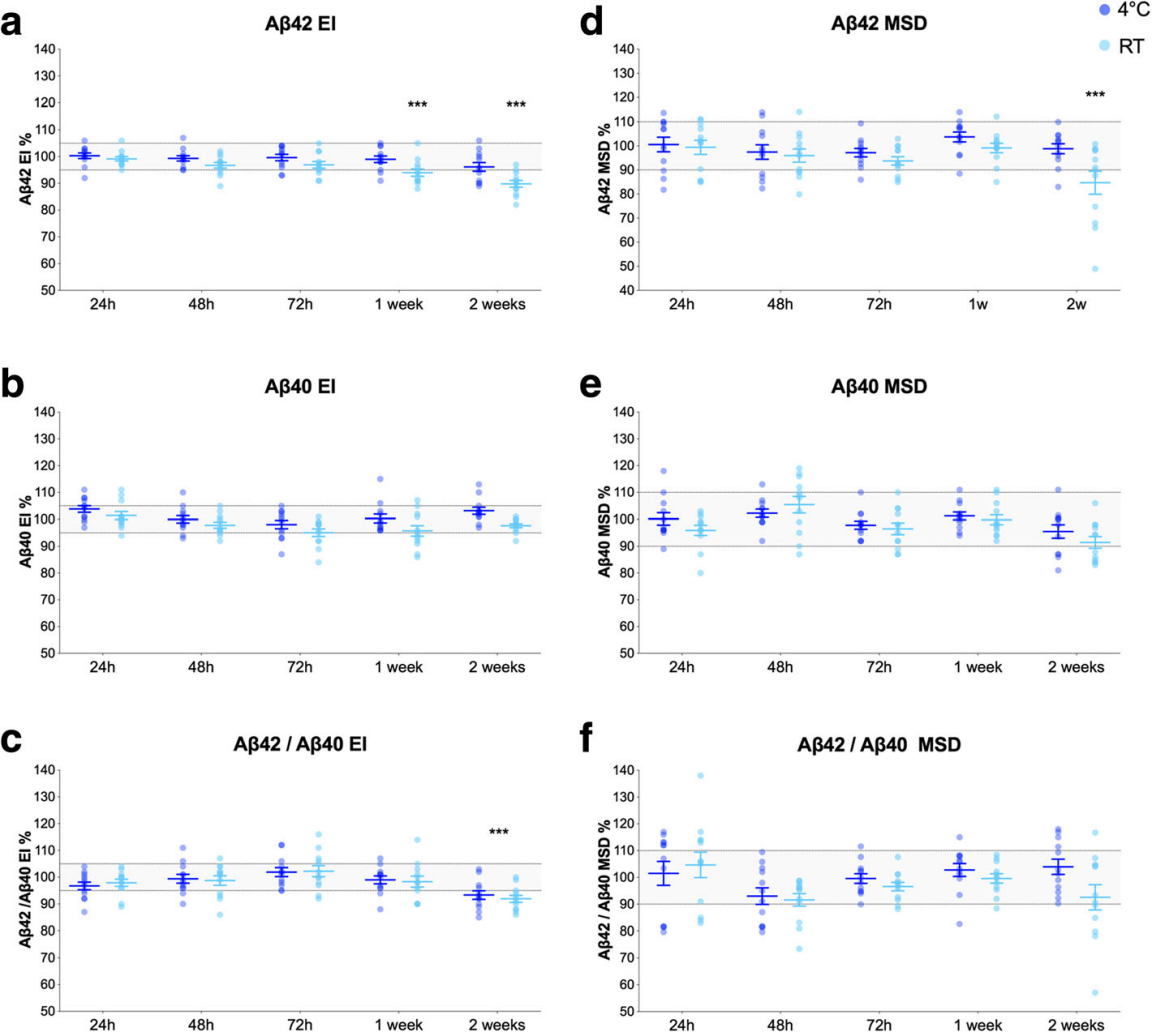
CSF storage at RT and 4 °C. Aβ42^EI^ (**a**), Aβ40^EI^ (**b**), Aβ42/Aβ40^EI^ (**c**), Aβ42^MSD^ (**d**), Aβ40^MSD^ (**e**), and Aβ42/Aβ40^MSD^ (**f**) in CSF samples that were stored at either RT or 4 °C for up to 2 weeks after collection (12 patients, 10 CSF tubes per patient). Data are shown as the percentage of biomarker levels in fresh, non-processed, CSF samples (from the same donor) that were analyzed within hours after LP. The effects of pre-analytical factors were tested using the mixed-effects model including participant identification as a random effect and temperature, time, and time × temperature interactions as fixed factors. The gray areas represent 95–105% and 90–110% ranges for the EI and MSD assays, respectively, that were set based on the inter-assay CVs as described in the “Materials and methods” section. Only changes in the mean biomarker levels outside these ranges (gray areas) were considered to be due to pre-analytical sample handling and examined using post hoc tests. *p ≤ 0.05, **p ≤ 0.01, ***p ≤ 0.001. Horizontal lines and error bars represent mean ± SEM. Abbreviations: Aβ, β-amyloid; EI, EUROIMMUN; h, hours; MSD, mesoscale discovery; RT, room temperature; SEM, standard error of mean

We sought to established optimal temperatures for CSF storage after collection and before Aβ analysis. Aβ42 and Aβ40 were measured on the day of CSF collection (baseline) and after storage at either RT or 4 °C for 24 h, 48 h, 72 h, 1 week, and 2 weeks (Fig. 1). In the mixed-effect model for Aβ42^EI^ (marginal and conditional *R*^2^ values of 0.004 and 0.995, respectively), there was a significant time × temperature interaction (*F* = 4.6, *p* < 0.001), and therefore, we performed simple main affects analysis for changes in the biomarker levels that exceeded 5%. In samples kept at 4 °C, Aβ42^EI^ levels were within 95–105% range of baseline at all time points (Fig. 1a). However, for samples stored at RT, the Aβ42^EI^ levels decreased by 6% after 1 week (*p* < 0.001) and 10% after 2 weeks (*p* < 0.001). Aβ40^EI^ levels were within 95–100% range of baseline at both temperatures and all time points (Fig. 1b) and were not tested in statistical analysis. For Aβ42/Aβ40^EI^, we found a significant effect of time (*F* = 10.3, *p* < 0.001) but no significant time × temperature interaction (marginal and conditional *R*^2^ values of 0.005 and 0.987, respectively). Aβ42/Aβ40^EI^ decreased by 8% and 7% when samples were stored for 2 weeks at RT and 4 °C, respectively (*p* < 0.001; Fig. 1c). The results were similar for the MSD assays (Fig. 1d–f). For baseline biomarker measurements, CSF samples from 10 different tubes per patients were analyzed on the day of LP. The mean CV values for these baseline measurements (10 tubes per patient, 12 patients) were 2.0%, 2.6%, 2.8%, 3.0%, 4.1, and 4.3% for Aβ42^EI^, Aβ40^EI^, Aβ42/Aβ40^EI^, Aβ42^MSD^, Aβ40^MSD^, and Aβ42/Aβ40^MSD^, respectively.

**Fig. 2:**
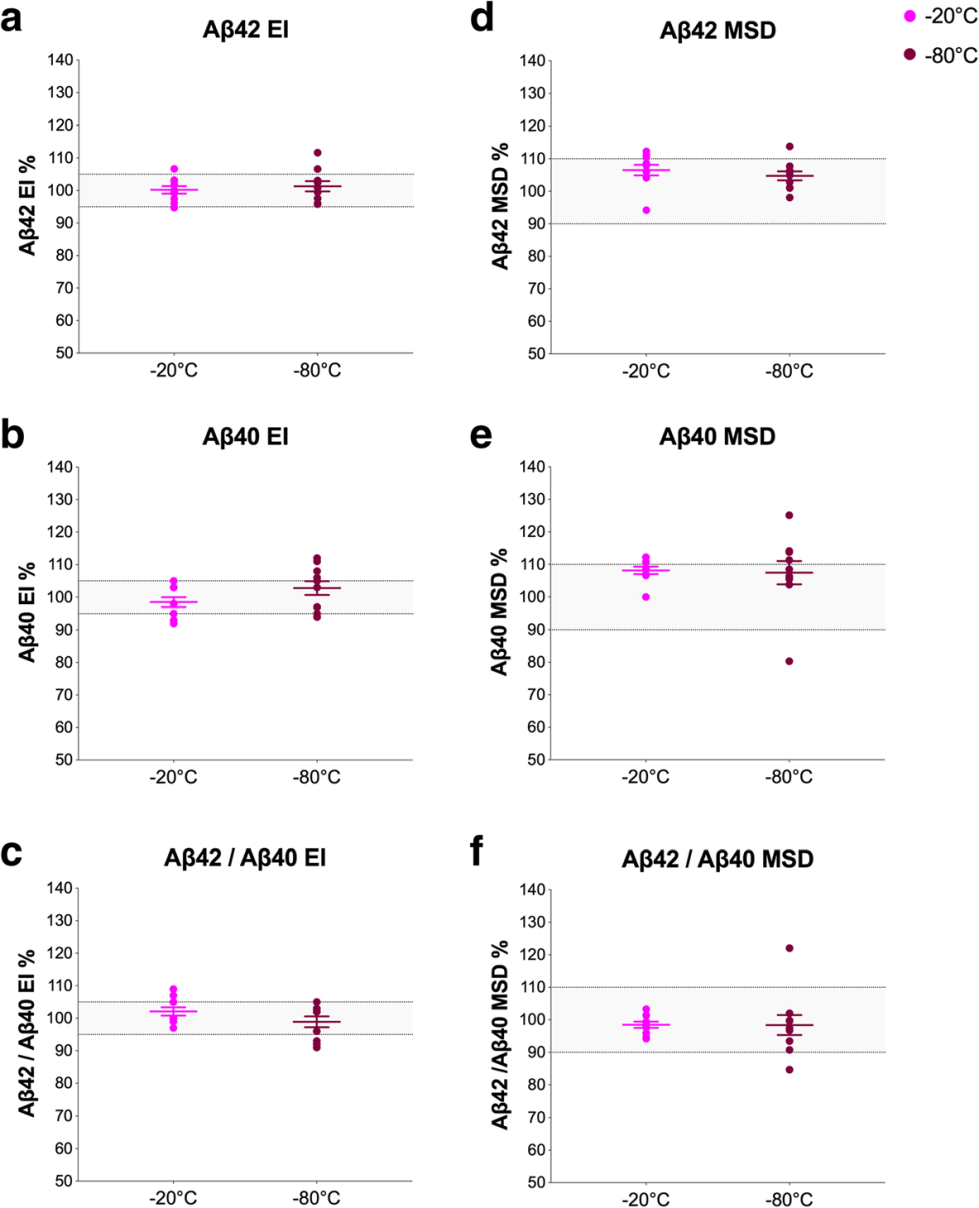
CSF storage at − 20 °C and − 80 °C. Aβ42^EI^ (**a**), Aβ40^EI^ (**b**), Aβ42/Aβ40^EI^ (**c**), Aβ42^MSD^ (**d**), Aβ40^MSD^ (**e**), and Aβ42/Aβ40^MSD^ (**f**) in CSF samples that were stored at either − 20 °C or − 80 °C for 2 weeks after collection (10 patients, 4 CSF tubes per patient). Data are shown as the percentage of biomarker levels in fresh, non-processed, CSF samples (from the same donor) that were analyzed within hours after LP. The gray areas represent 95–105% and 90–110% ranges for the EI and MSD assays, respectively, that were set based on the inter-assay CVs as described in the “Materials and methods” section. Changes in the mean biomarker levels were all within 95–105% range of baseline at both temperatures and, therefore, were not tested in the statistical analysis. Horizontal lines and error bars represent mean ± SEM. Abbreviations: Aβ, β-amyloid; EI, EUROIMMUN; MSD, Mesoscale discovery; SEM, standard error of mean

Next, we compared Aβ concentrations in CSF samples that were stored at − 20 °C or − 80 °C for 2 weeks after collection. Aβ42^EI^, Aβ40^EI^, and Aβ42/Aβ40^EI^ were all within 95–105% range of baseline at both temperatures (Fig. 2a–c). The results were similar for the MSD assays (Fig. 2d–f).

### Mixing of CSF before Aβ measurements

**Fig. 3:**
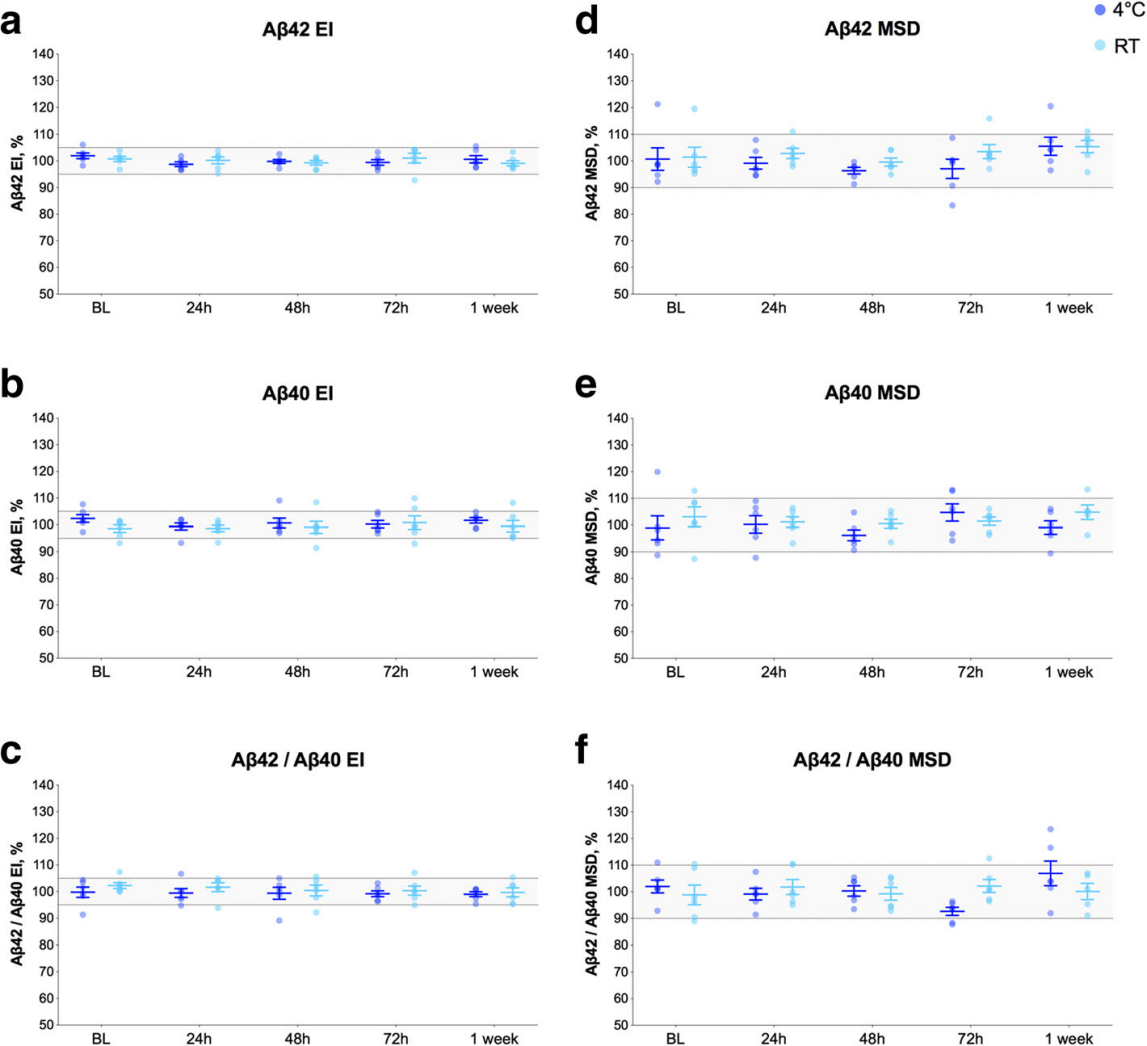
Mixing of CSF before Aβ measurements. Aβ42^EI^ (**a**), Aβ40^EI^ (**b**), Aβ42/Aβ40^EI^ (**c**), Aβ42^MSD^ (**d**), Aβ40^MSD^ (**e**), and Aβ42/Aβ40^MSD^ (**f**) in CSF samples that were not mixed before Aβ measurements after being stored at either RT or 4 °C for up to 1 week after collection (6 patients, 4 CSF tubes per patient). Data are shown as the percentage of biomarker levels in CSF samples (from the same donor) that were mixed for 15 min using a roller mixer immediately before the analysis and treated the same way with respect to other experimental conditions (temperature and time). The gray areas represent 95–105% and 90–110% ranges for the EI and MSD assays, respectively, that were set based on the inter-assay CVs as described in the “Materials and methods” section. All biomarker levels in unmixed CSF samples were within 95–105% range of the mixed samples at both temperature and all time points and, therefore, were not tested in the statistical analysis. Horizontal lines and error bars represent mean ± SEM. Abbreviations: Aβ, β-amyloid; BL, baseline; EI, EUROIMMUN; h, hours; MSD, Mesoscale discovery; RT, room temperature; SEM, standard error of mean

Here, we investigated if it is necessary to mix CSF samples prior to Aβ measurements. Aβ42 and Aβ40 concentrations were determined in mixed and unmixed CSF samples on the day of collection and after storage at either RT or 4 °C for 24 h, 48 h, 72 h, and 1 week. Aβ42^EI^, Aβ40^EI^, and Aβ42/Aβ40^EI^ in unmixed CSF samples were within 95–105% range of the mixed samples at both temperature and all time points (Fig. 3a–c). The results were similar for the MSD assays (Fig. 3d–f).

For frozen CSF samples that were not mixed after thawing, we observed high variability in the mean change from baseline (Aβ42 CV = 15.5%) compared to mixed sample (Aβ42 CV = 2.7%).

### Centrifugation and blood contamination

We also assessed the effects of centrifugation after LP and/or blood contamination on Aβ levels in CSF stored at different temperatures for 24 h, 72 h, 1 week, and 2 weeks after collection.

First, we investigated the effect of centrifugation on Aβ levels in neat (without added blood) CSF. We did not observe differences in the biomarker levels between neat centrifuged and non-centrifuged CSF samples stored for up to 2 weeks at RT, 4 °C, or − 20 °C (Additional file 1: Figure S8).

**Fig. 4:**
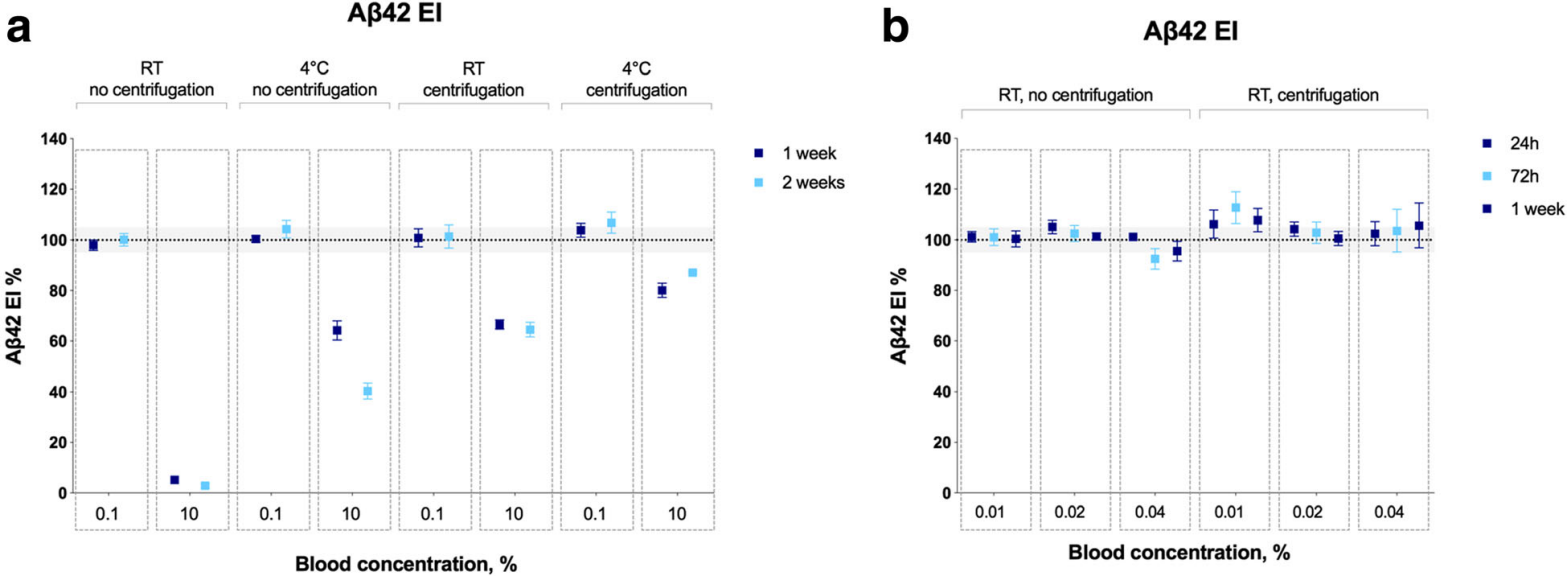
Blood contamination—EI assay. **a** Aβ42^EI^ in CSF samples with added 0.1% or 10% blood stored at either RT or 4 °C for up to 2 weeks after collection (4 patients, 12 CSF tubes per patient; except the 10% blood, 4 °C, centrifugation group from where one sample was excluded due to the technical error during the Aβ42 measurements). **b** Aβ42^EI^ in CSF samples with added 0.01%, 0.02, or 0.04% blood stored at RT for up to 1 week after collection (4 patients, 8 CSF tubes per patient). Data are shown as the percentage of biomarker levels in neat CSF samples (from the same donor) that were treated the same way with respect to other experimental conditions (centrifugation, temperature, and time). The gray areas represent 95–105% range that was set based on the inter-assay CVs as described in the “Materials and methods” section. Given the small number of participants, statistical tests were not performed. Horizontal lines and error bars represent mean ± SEM. Abbreviations: Aβ, β-amyloid; EI, EUROIMMUN; RT, room temperature; SEM, standard error of mean

**Fig. 5:**
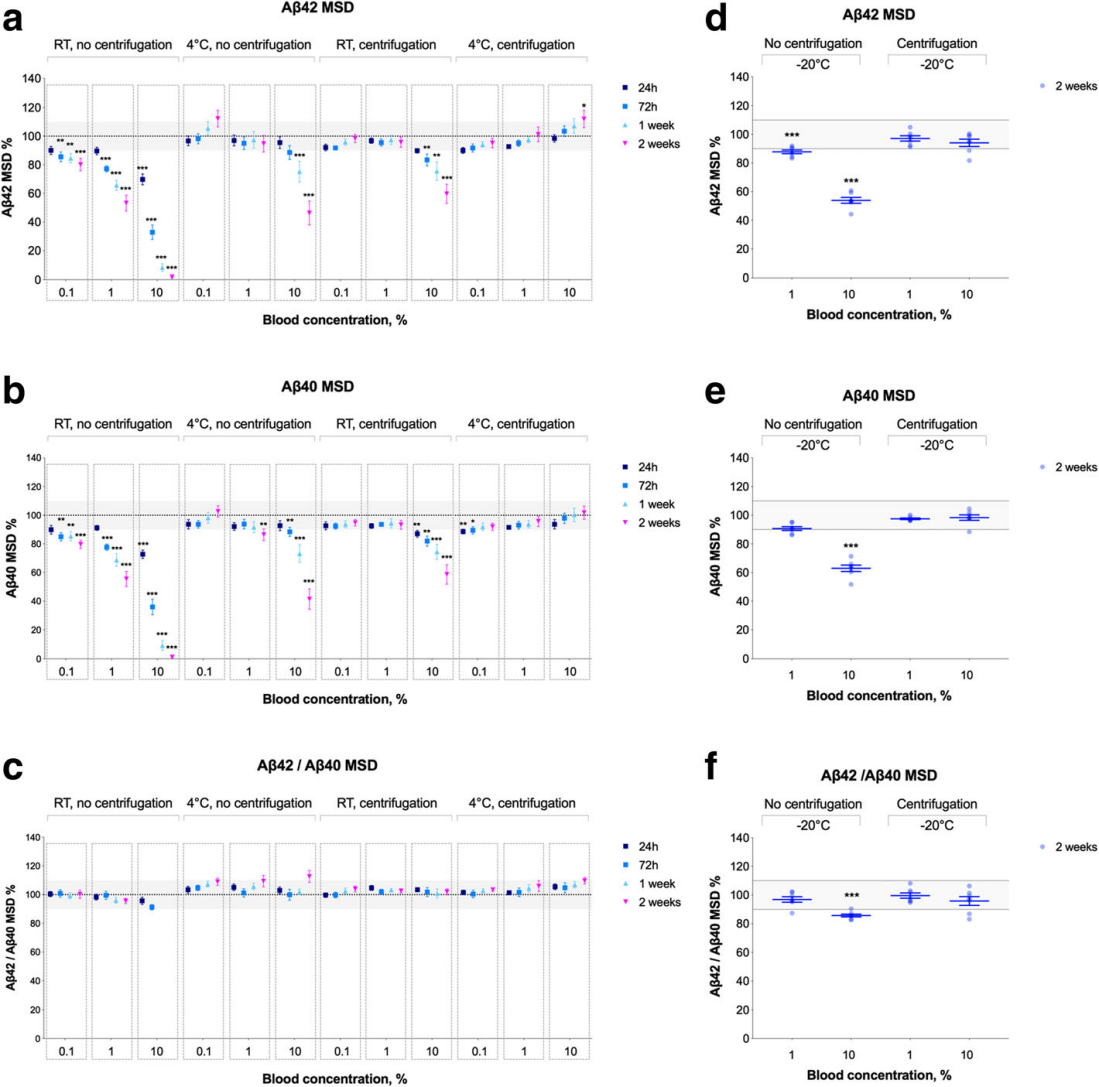
Blood contamination—MSD assays. Aβ42^MSD^ (**a**), Aβ40^MSD^ (**b**), and Aβ42/Aβ40^MSD^ (**c**) in CSF samples with added blood stored at either RT or 4 °C for up to 2 weeks after collection. Aβ42^MSD^ (**d**), Aβ40^MSD^ (**e**), and Aβ42/Aβ40^MSD^ (**f**) in CSF samples with added blood stored at − 20 °C for 2 weeks after collection. CSF was collected from 7 to 10 patients per experimental condition, 24 CSF tubes per patients. Data are shown as the percentage of biomarker levels in neat CSF samples (from the same donor) that were treated the same way with respect to other experimental conditions (centrifugation, temperature, and time). The effects of pre-analytical factors were tested using mixed-effects model including participant identification as a random effect and treatment groups (temperature, centrifugation, and blood contamination), time, and time × treatment group interactions as fixed factors. The gray areas represent 90–110% range that was set based on the inter-assay CVs as described in the “Materials and methods” section. Only changes in the mean biomarker levels outside this range (gray area) were considered to be due to pre-analytical sample handling and examined using post hoc tests. *p ≤ 0.05, **p ≤ 0.01, ***p ≤ 0.001. **c** Because of the very low levels of CSF Aβ42 and Aβ40 (< 10 pg/ml), we did not calculate the Aβ42/Aβ40 ratio for non-centrifuged CSF-10%-blood samples that were stored at RT for 1–2 weeks. Horizontal lines and error bars represent mean ± SEM. Abbreviations: Aβ, β-amyloid; EI, EUROIMMUN; h, hours; MSD, Mesoscale discovery; RT, room temperature; SEM, standard error of mean

Next, we studied the effects of adding fresh blood to fresh CSF. In the initial experiments, we examined the effects of 0.1% and 10% blood on CSF Aβ42^EI^ values over 2 weeks and observed dramatic effects of 10% blood contamination on Aβ42^EI^ levels, which was partly mitigated by centrifugation and/or storage at 4 °C (Fig. 4a). Because we found an effect of blood contamination on CSF Aβ42, we made more detailed analyses assessing how 0.1%, 1.0%, and 10% blood contamination affects the CSF levels of Aβ42^MSD^, Aβ40^MSD^, and Aβ42/Aβ40^MSD^ over 2 weeks of storage. In the mixed-effect model for Aβ42 (marginal and conditional *R*^2^ values of 0.309 and 0.890, respectively) and Aβ40 (marginal and conditional *R*^2^ values of 0.311 and 0.877, respectively), there were significant time × treatment group interactions (*F* = 2.2–27.0, *p* < 0.001), and therefore, we performed simple main affects analysis for changes in the biomarker levels exceeding 5%. Again, we found that blood contamination led to a dose- and time-dependent decrease (by 14–98%) in Aβ42 and Aβ40 (Fig. 5a, b). Interestingly, Aβ42/Aβ40 ratio did not change in CSF samples that were stored at RT without centrifugation (Fig. 5c). The effects of blood contamination on CSF Aβ42 and Aβ40 levels were partially mitigated by centrifugation or storage at 4 °C, and when combining centrifugation with storage at 4 °C, there were no effects even when adding 10% blood. Further, centrifugation and storage at − 20 °C effectively blocked the effects of up to 10% blood contamination on CSF Aβ42 and Aβ40 levels (Fig. 5d–f).

The largest reduction in CSF levels of Aβ was seen for non-centrifuged samples stored at room temperature. Therefore, we further investigated the effects of blood contamination at levels below 0.1% on CSF Aβ42 under these conditions. Although blood contamination at 0.04% (2000 E/μl) was still visible by the unaided eye, CSF-0.02%-blood (1000 E/μl) and CSF-0.01%-blood (500 E/μl) samples were visually indistinguishable from neat CSF (Additional file 1: Figure S4). In non-centrifuged CSF-0.01%-blood and CSF-0.02%-blood samples, Aβ42 levels were stable for up to 1 week after collection (Fig. 4b). However, the addition of 0.04% blood caused a small (5–8%) decrease in Aβ42 at 72 h and 1 week after collection which was again blocked by centrifugation (Fig. 4b).

### Collection gradient

**Fig. 6:**
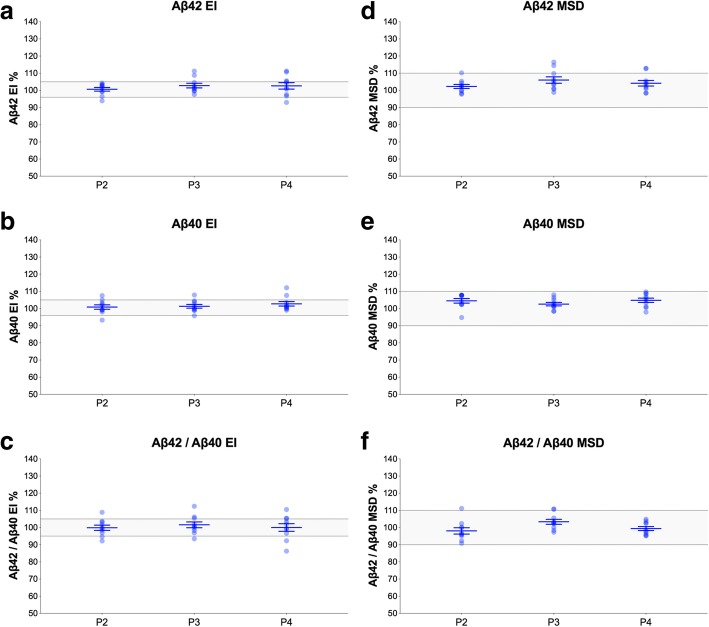
CSF collection gradient. Aβ42^EI^ (**a**), Aβ40^EI^ (**b**), Aβ42/Aβ40^EI^ (**c**), Aβ42^MSD^ (**d**), Aβ40^MSD^ (**e**), and Aβ42/Aβ40^MSD^ (**f**) in the second (P2), third (P3), and fourth (P4) 5-ml portions of 20 ml CSF collected after discarding the first 2 ml (10 patients). Data are shown as the percentage of biomarker levels in the first portion. The gray areas represent 95–105% and 90–110% ranges the EI and MSD assays, respectively, that were set based on the inter-assay CVs as described in the “Materials and methods” section. The mean biomarker levels in the second to fourth fractions were all within 95–105% range of the first fractions and, therefore, were not tested in statistical analysis. Horizontal lines and error bars represent mean ± SEM. Abbreviations: Aβ, β-amyloid; EUROIMMUN; MSD, Mesoscale discovery; P, portion; SEM, standard error of mean

To determine whether the caudal-rostral concentration gradient has an effect on CSF Aβ, we measured Aβ42 and Aβ40 in the first to fourth 5-ml fractions of 20 ml CSF collected during LP. When compared with the first fraction, Aβ42^EI^, Aβ40^EI^, and Aβ42/Aβ40^EI^ were not altered by more than 5% in any of the other fractions (Fig. 6a–c). The results were similar for the MSD assays (Fig. 6d–f).

## Discussion

The aim of the present study was to perform experiments needed to facilitate the development of a unified standard operating procedure for pre-analytical handling of CSF optimized for Aβ measurements. To this end, we investigated the effects of a number of pre-analytical factors on CSF levels of Aβ42 and Aβ40 using fresh (non-processed) CSF samples analyzed within hours after LP as the standard of truth. Our findings indicate that CSF could be stored for at least 72 h at RT, 1 week at 4 °C, and 2 weeks at − 20 °C and − 80 °C without significant changes in the levels of Aβ42 or Aβ40. Centrifugation was not needed if the CSF samples were not contaminated with blood. Further, we found that there is no need to mix the CSF samples prior to Aβ measurements, if the samples had not been frozen. In CSF samples that were not centrifuged after LP and stored at RT, blood contamination led to dose- and time-dependent reductions in Aβ42 and Aβ40 levels whereas Aβ42/Aβ40 was stable. The effects of blood contamination on Aβ42 and Aβ40 were partially to fully mitigated by centrifugation and/or storage at lower temperatures (4 °C and − 20 °C). Finally, we did not observe differences in Aβ42 and Aβ40 levels between the first and second to fourth 5-ml portions of 20 ml CSF collected during LP. Although we used LoB tubes from Sarstedt in the present study, our unpublished data indicates no difference in Aβ levels between CSF samples stored in either Sartstedt or Eppendorf LoB tubes. In principle, LoB tubes available from other vendors could also be suitable for CSF storage before Aβ analysis. Still, their performance should be assessed in comparison to Sartstedt or Eppendorf LoB tubes before incorporation in pre-analytical protocols.

For practical reasons, it is more suitable to transport CSF to clinical chemistry laboratories for testing at RT or at 4 °C. However, at these temperatures, Aβ42 release from amyloid-binding proteins, adsorption to tube material, or proteolytic degradation may all potentially cause changes in the levels of this peptide [8], particularly in CSF samples stored for several days or longer. Published evidence indicates that Aβ42 is stable when CSF is stored at RT for up to 24 h [12, 19, 20]. At the same time, data on longer storage have been inconclusive. Some reports have shown that storage for 2–14 days does not affect CSF levels of Aβ42 levels [20–22], while others found both an increase [12] and decrease [23] in Aβ42 concentrations by 15–20%. Using LoB tubes and fresh (non-processed) CSF analyzed within hours after LP as the standard of truth, we observed that Aβ42 and Aβ40 levels were stable for up to 2 weeks and Aβ42/Aβ40 for up to 1 week when samples were stored at 4 °C. Storage at RT led to 6–10% decline in Aβ42 and Aβ42/Aβ40 at 1 and 2 weeks after collection. Collectively, these results indicate that CSF samples could be stored for at least 72 h at RT and 1 week at 4 °C without significant changes in Aβ. Thus, in these conditions, release from amyloid-binding proteins and proteolytic degradation may have limited effects on CSF levels of Aβ42. Of note, the observed changes in the biomarker levels after 72 h and 1 week were small. Nevertheless, storage of CSF samples longer than these time periods might not be recommended considering that (i) experimental conditions (e.g., temperature) in the present study were tightly controlled which would be difficult to implement in routine clinical practice worldwide and (ii) for biomarker levels that are close to the cutoffs for Aβ abnormality even small changes due to pre-analytical handling could have significant impact on diagnostic process. Corroborating previous data [12], we further demonstrate no differences in Aβ42 and Aβ40 between samples stored at − 20 °C and− 80 °C for 2 weeks and, therefore, either of the temperatures could be used if freezing of samples before analyses is preferred.

Freezing and thawing procedures introduce concentration gradient in biological samples, and consequently, thorough mixing is needed to make samples homogeneous [24] Accordingly, we found that analysis of unmixed freeze-thawed CSF samples produced unreliable Aβ values with high variability. However, it is not established whether fresh CSF samples should be mixed prior to Aβ measurements, especially after relatively longer storage at RT or 4 °C. In the present study, we observed no differences in Aβ42 and Aβ40 between mixed and unmixed fresh CSF samples that were stored at either RT or 4 °C for up to 1 week after collection. Similarly, one earlier study has shown that vortexing of CSF did not have any significant effects on Aβ42 level [25]. Thus, for CSF samples stored at RT or 4 °C, mixing prior to Aβ measurements is not required.

Centrifugation of CSF after LP is usually advised to remove white blood cells and, in case of blood contamination, red blood cells that may alter the proteome profile [26–28]. Yet, there is a lack of evidence about the effects of centrifugation on Aβ42 levels in CSF stored at RT or 4 °C for longer than 24 h, while data for frozen CSF samples are not consistent. Previous studies have shown both no change and a reduction in CSF Aβ42 after centrifugation [12, 19, 23, 29–31]. However, all of these studies used polypropylene tubes for CSF collection, and only one examined the effects of centrifugation in fresh CSF samples. Thus, a drop in Aβ42 levels due to adsorption to the tube walls and freeze-thaw cycles might have confounded the reported results. Here, using the LoB tube and fresh CSF analyzed within hours after LP as the standard of truth, we demonstrate that centrifugation after LP does not affect Aβ42 and Aβ40 levels in non-hemorrhagic CSF samples stored at RT, 4 °C, and − 20 °C for up to 2 weeks after collection. Hence, for CSF samples with no visible blood contamination, centrifugation is not necessary.

In agreement with previous data [19], we observed that blood contamination alters CSF levels of Aβ. Addition of 0.04–10% of blood corresponding to 2 × 10^3^–5 × 10^5^ E/μl to CSF samples caused a 5–98% decline in Aβ42 and Aβ40 concentrations which was more pronounced and occurred earlier with increasing degree of blood contamination. At this level, blood contamination was visible by the unaided eye (Additional file 1: Figure S4). Notably, centrifugation and/or storage at lower temperatures (4 °C and − 20 °C) mitigated the effects of blood contamination. A possible explanation for these findings could be that centrifugation and low temperatures attenuate the binding and degradation of CSF Aβ peptides by blood-derived proteins that have been suggested to affect the biomarker levels in blood-contaminated samples [8]. The magnitude of decrease in CSF Aβ42 and Aβ40 concentrations was similar, and consequently, Aβ42/Aβ40 was not significantly influenced by blood contamination. In line with our data, previous investigations have demonstrated that Aβ42/Aβ40 showed improved accuracy for AD diagnosis [2] and superior concordance with amyloid PET [32–35] in part by normalizing variability due to pre-analytical factors [11, 25, 36, 37]. We did not observe any effects of blood contamination at levels that were not visible by the unaided eye (0.01% [500 E/μl] and 0.02% [1000 E/μl]) on CSF Aβ42. Taken together, our findings suggest that for CSF samples with more than 1000 E/μl blood contamination or visible blood contamination (if erythrocyte count is not available), centrifugation and storage at 4 °C or − 20 °C should be recommended.

Some proteins show differences in concentrations between lumbar and ventricular CSF, suggesting the existence of rostro-caudal concentration gradient [38–40]. Such a gradient would imply that biomarker levels vary depending on the volume and fraction of CSF collected during LP. Speaking against rostro-caudal concentration gradient for Aβ and in agreement with previous studies, we did not observe any differences in Aβ42 and Aβ40 levels between the first to fourth 5-ml portions of CSF.

One limitation of the present study is bias associated with inter-assay variability. For example, in the case of the EI assays, we observed 5% fluctuations in the biomarker concentrations due to run-to-run variance in assay performance. Consequently, we were unable to reliably detect changes caused by pre-analytical factors that were below 5%.

## Conclusions

The conclusions of the present study are as follows: (1) any portion of up to 20 ml of CSF (after discarding the first 2 ml) could be used for Aβ analysis, (2) CSF should be collected directly into a LoB tube, (3) centrifugation of CSF after LP is not necessary if erythrocyte count is ≤ 1000/μl or no visible blood contamination, (4) fresh CSF samples could be stored at RT for 72 h or at 4 °C for 1 week until analyses, (5) mixing of fresh CSF samples before Aβ measurement is not required, and (6) CSF samples with visible blood contamination should be centrifuged after LP and stored at 4 °C. An alternative approach is to store the CSF collected directly into a LoB tube at − 20 °C or − 80 °C until analyses. The frozen samples should be handled similar to the fresh samples (see above), except that mixing of the thawed samples should be done just before analyses. Although the findings of the present study were very similar for EI and MSD assays, they should be verified for other available Aβ assays/platforms. A final unified pre-analytical protocol must be decided in consensus by the main stakeholders in the field. This effort is currently led by the Alzheimer’s Association*.*

## Additional file

Additional file 1: **Figure S1.** Flowchart of the CSF storage protocol. **Figure S2.** Flowchart of the CSF mixing protocol. **Figure S3.** Flowchart of the blood contamination (0.1%, 1%, 10%) and centrifugation protocol—EI assay. **Figure S4.** CSF-blood samples. **Figure S5.** Samples and analysis in blood contamination and centrifugation protocol—MDS assay. **Figure S6.** Flowchart of blood contamination at low levels (0.01%, 0.02%, 0.04%) and the centrifugation protocol—EI assay. **Figure S7.** Frequency plots of CSF biomarkers. **Figure S8.** Effects of centrifugation when not adding blood. (DOCX 8700 kb)

## Data Availability

Anonymized data will be shared by request from any qualified investigator for the sole purpose of replicating procedures and results presented in the article and as long as data transfer is in agreement with EU legislation on the general data protection regulation.

## Abbreviations

AD: Alzheimer’s disease
Aβ: β-Amyloid
CSF: Cerebrospinal fluid
CV: Coefficient of variance
EI: EUROIMMUN
LoB: Low binding
LP: Lumbar puncture
MSD: Mesoscale discovery
P: Portion
RT: Room temperature
