# Supplementary material for: Towards a unified protocol for handling of CSF before β-amyloid measurements
Source: Alzheimers Res Ther. 2019 Jul 19;11:63. doi: 10.1186/s13195-019-0517-9 (PMC6642586; doi:10.1186/s13195-019-0517-9)
Supplement: Supplementary file 1 — Figure S1. Flowchart of the CSF storage protocol. Figure S2. Flowchart of the CSF mixing protocol. Figure S3. Flowchart of the blood contamination (0.1%, 1%, 10%) and centrifugation protocol—EI assay. Figure S4. CSF-blood samples. Figure S5. Samples and analysis in blood contamination and centrifugation protocol—MDS assay. Figure S6. Flowchart of blood contamination at low levels (0.01%, 0.02%, 0.04%) and the centrifugation protocol—EI assay. Figure S7. Frequency plots of CSF biomarkers. Figure S8. Effects of centrifugation when not adding blood. (DOCX 8700 kb) [file 13195_2019_517_MOESM1_ESM.docx]

**ADDITIONAL FILE 1**

**Towards a unified protocol for handling of CSF before β-amyloid measurements**

Shorena Janelidze PhD, Erik Stomrud, MD, PhD, Britta Brix, PhD, and Oskar Hansson MD, PhD

**Figure S1. Flowchart of the CSF storage protocol.**

CSF samples were collected from 12 individuals in (A) and from additional 10 individuals in (B). Each flowchart shows a protocol for individual patient. Samples from different patients within the same protocol were treated the same way. * T1 And T2 from 6 individuals in (B) were not mixed prior to the Aβ analysis.

**Figure S2. Flowchart of the CSF mixing protocol.**

CSF samples were collected from 6 individuals. Each flowchart shows a protocol for individual patient. Samples from different patients were treated the same way.

**Figure S3. Flowchart of the blood contamination (0.1%, 1%, 10%) and centrifugation protocol – EI assay.**

CSF samples were collected from 4 individuals. Each flowchart shows a protocol for individual patient. Samples from different patients were treated the same way.

**Figure S4. CSF-blood samples.**

Blood (final volume 0.01, 0.02, 0.04, 0.1%, 1% or 10%) was added to CSF samples from the same individual within immediately after of collection.

**
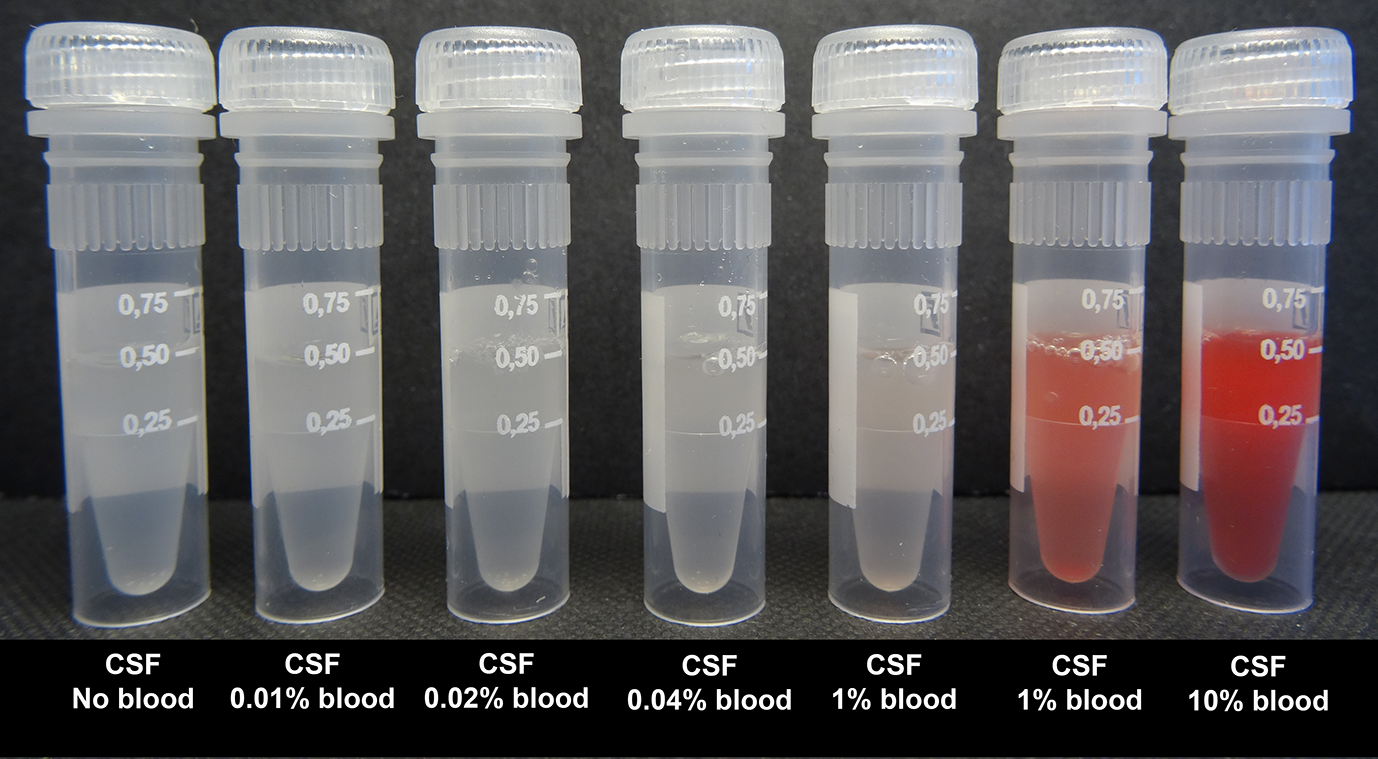
**

**Figure S5. Samples and analysis in blood contamination and centrifugation protocol – MDS assay.**

CSF samples were collected from 7-10 individuals per experimental condition.

**Figure S6. Flowchart of blood contamination at low levels (0.01%, 0.02%, 0.04%) and centrifugation protocol – EI assay.**

CSF samples were collected from 4 individuals. Each flowchart shows a protocol for individual patient. Samples from different patients were treated the same way.

**Figure S7. Frequency plots of CSF biomarkers.**

Histograms of frequency distribution for CSF Aβ42^EI^ (**A**), Aβ40^EI^ (**B)**, Aβ42/Aβ40^EI^ (**C**), Aβ42^MSD^ (**D**), Aβ40^MSD^(**E**) and Aβ42/Aβ40^MSD^ (**F**)**.**

**
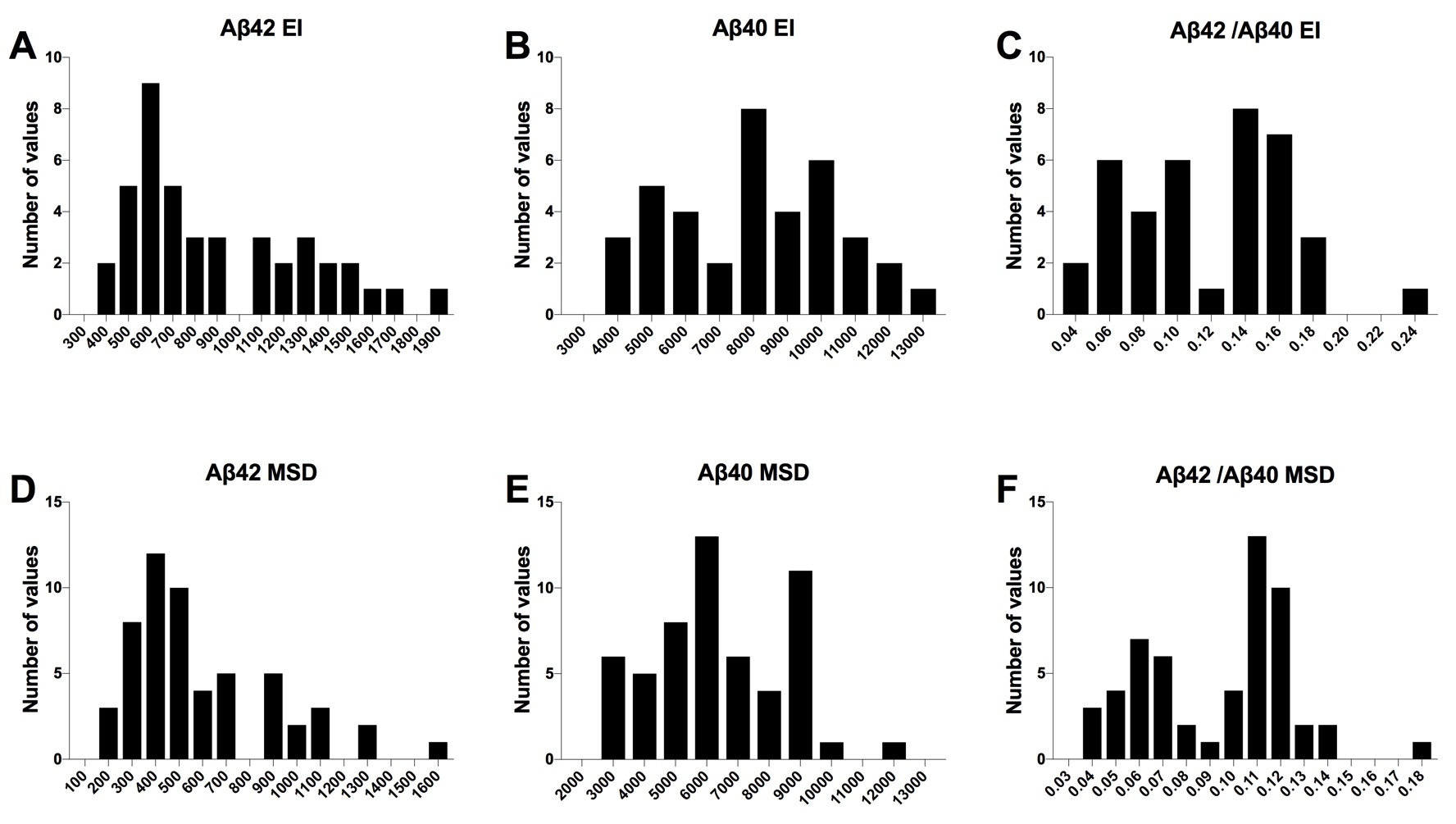
**

**Figure S8. Effects of centrifugation when not adding blood.**

Aβ42^EI^ (**A**), Aβ42^MSD^ (**B**), Aβ40^MSD^ (**C**) and Aβ42/Aβ40^MSD^ (**D**) in non-centrifuged CSF samples stored at either RT or 4°C for up to 2 weeks after collection (MSD n=10-17, EI n=5). Aβ42^MSD^ (**E**), Aβ40^MSD^ (**F**) and Aβ42/Aβ40^MSD^ (**G**) in non-centrifuged CSF samples stored at -20°C for 2 weeks after collection (n=7). Data are shown as percentage of biomarker levels in centrifuged CSF samples that were treated the same way with respect to other experimental conditions (temperature and time). The grey areas represent 90-110% and 95-105% ranges the MSD and EI assays, respectively, that were set based on the inter-assay CVs as described in the methods. When the mean biomarker levels in non-centrifuged samples were outside the respective ranges (grey areas), the differences between centrifuged and non-centrifuged samples were tested using paired sample T test (* p≤0.05, ** p≤0.01, *** p≤0.001). Abbreviations: Aβ, β-amyloid; BL, baseline; EI, EUROIMMUN; h, hours; MSD, Mesoscale discovery; RT, room temperature.

**
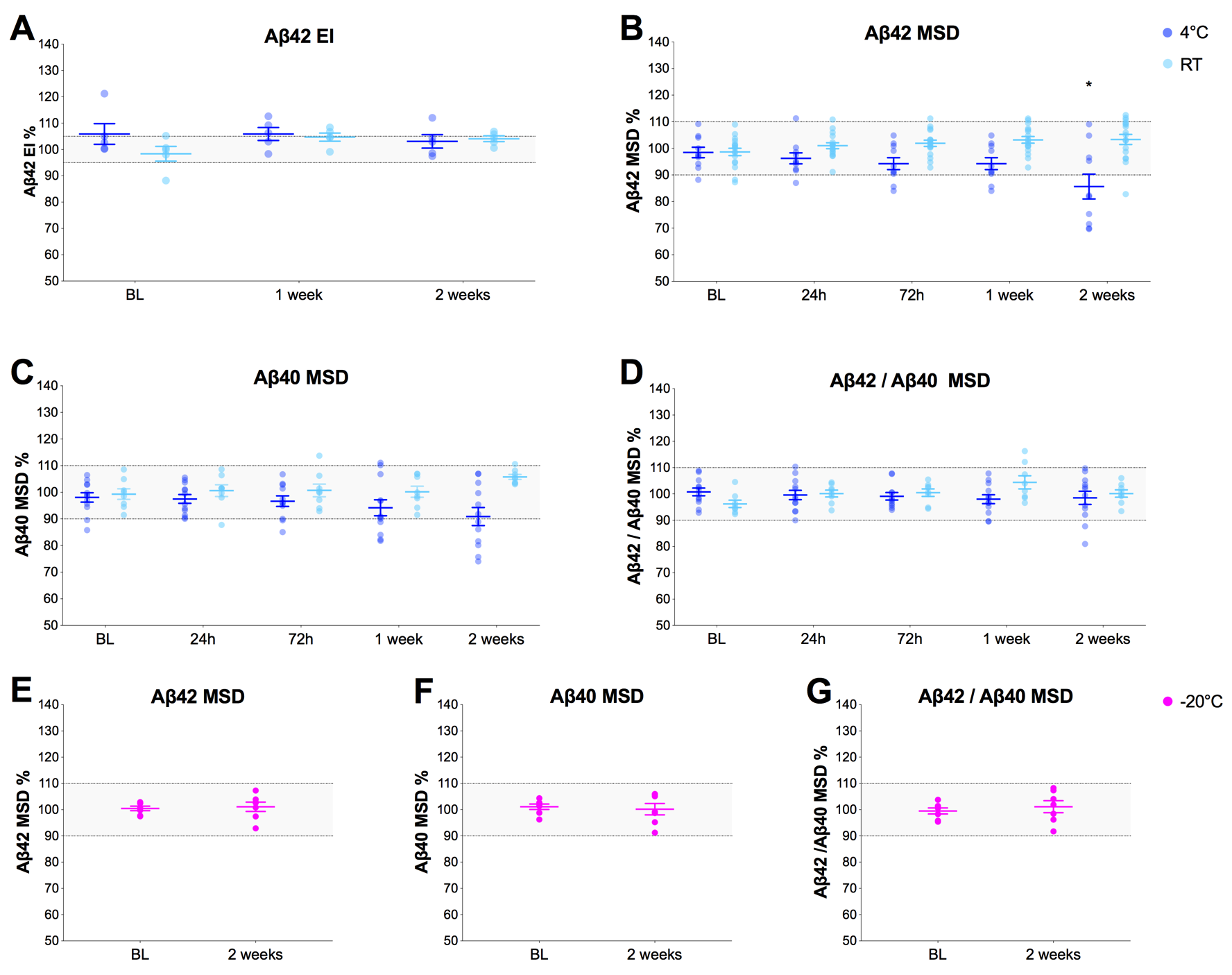
**
